# The mycobacterial proteasomal ATPase Mpa forms a gapped ring to engage the 20S proteasome

**DOI:** 10.1016/j.jbc.2021.100713

**Published:** 2021-04-27

**Authors:** Yanting Yin, Amanda Kovach, Hao-Chi Hsu, K. Heran Darwin, Huilin Li

**Affiliations:** 1 Department of Structural Biology, Van Andel Institute, Grand Rapids, Michigan, USA; 2 Department of Microbiology, New York University Grossman School of Medicine, New York, New York, USA

**Keywords:** mycobacterial proteasome ATPase, *Mycobacterium tuberculosis*, structural biology, cryo-EM, 20S_OG_, open-gate 20S, AAA, *A*TPase *A*ssociated with various *A*ctivities, AMP-PNP, adenylyl-imidodiphosphate, CP, core particle, FabD, malonyl CoA-acyl carrier protein transacylase, GQYL, Gly–Gln–Tyr–Leu, Mtb, *Mycobacterium tuberculosis*, OB, oligonucleotide-/oligosaccharide-binding, Paf, proteasome accessory factor, PAN, proteasome-activating nucleotidase, Pup, prokaryotic ubiquitin-like protein

## Abstract

Although many bacterial species do not possess proteasome systems, the actinobacteria, including the human pathogen *Mycobacterium tuberculosis*, use proteasome systems for targeted protein removal. Previous structural analyses of the mycobacterial proteasome ATPase Mpa revealed a general structural conservation with the archaeal proteasome-activating nucleotidase and eukaryotic proteasomal Rpt1–6 ATPases, such as the N-terminal coiled-coil domain, oligosaccharide-/oligonucleotide-binding domain, and ATPase domain. However, Mpa has a unique β-grasp domain that in the ADP-bound crystal structure appears to interfere with the docking to the 20S proteasome core particle (CP). Thus, it is unclear how Mpa binds to proteasome CPs. In this report, we show by cryo-EM that the Mpa hexamer in the presence of a degradation substrate and ATP forms a gapped ring, with two of its six ATPase domains being highly flexible. We found that the linkers between the oligonucleotide-binding and ATPase domains undergo conformational changes that are important for function, revealing a previously unappreciated role of the linker region in ATP hydrolysis–driven protein unfolding. We propose that this gapped ring configuration is an intermediate state that helps rearrange its β-grasp domains and activating C termini to facilitate engagement with proteasome CPs. This work provides new insights into the crucial process of how an ATPase interacts with a bacterial proteasome protease.

Proteasomes are found in all eukaryotes and archaea (1, 2) and in two orders of bacteria, the *Actinomycetales* and *Nitrospirales* (3). The human pathogen *Mycobacterium tuberculosis* (Mtb) is an actinobacterial species and requires a proteasome for survival in a mammalian host (4, 5). The bacterial proteasome core particle (20S CP) is similar to archaeal and eukaryotic 20S CPs, in which 14 proteasome core α-subunits and 14 proteasome core β-subunits form a four-ring stacked cylinder (α_7_β_7_β_7_α_7_) with 14 proteolytic Thr-1 residues of the β-subunits enclosed inside a chamber (6). The entry gate of an Mtb 20S CP is largely closed and requires accessory factors to open it for substrate delivery (6, 7). The bacterial system is conceptually parallel to the eukaryotic ubiquitin–proteasome pathway, in which ubiquitylated proteins are recognized, unfolded, and delivered into 20S CPs for degradation by a regulatory structure that includes at its base a heterohexameric ATPase ring (1, 8). However, in Mtb, the accessory factors that aid proteolysis are distinct from those found in eukaryotes and archaea (9). In particular, actinobacteria like Mtb use a post-translational modification called prokaryotic ubiquitin-like protein (Pup), which looks nothing like eukaryotic ubiquitin (9, 10). Pup is a mostly disordered small protein (64 amino acids in Mtb) ending in Gln (Q), which needs to be converted to Glu (E) by the deamidase of Pup before it can be covalently linked to a lysine in a target protein by the ligase proteasome accessory factor (Paf) A in an ATP-dependent mechanism (11). Pupylated substrates are recognized, unfolded, and delivered into 20S CPs for degradation by the homohexameric ATPase Mpa (also known as ATPase forming ring-shaped complex in other genera) (12, 13, 14, 15, 16, 17).

Mpa is the most well-characterized bacterial proteasomal ATPase. It has an N-terminal coiled-coil domain responsible for Pup recognition (14, 18); an intermediate region with two oligonucleotide-/oligosaccharide-binding (OB) domains that promote hexamer assembly and structural stability (19); a canonical *A*TPase *A*ssociated with various *A*ctivities (AAA) domain with an internal pore loop that binds and translocates polypeptides; and a C-terminal β-grasp domain (20) (Fig. 1, *A* and *B*). Instead of a “hydrophobic-tyrosine-X” motif found in the archaeal and eukaryotic proteasomal ATPases (21, 22, 23), Mpa has a C-terminal Gly–Gln–Tyr–Leu (GQYL) motif that is required for opening the substrate entry gate of 20S CPs (24). Mpa structurally resembles the archaeal proteasome activator proteasome-activating nucleotidase (PAN) and the eukaryotic Rpt1–6 ATPase activator ring, in that they all contain a coiled-coil domain, an OB fold, and an AAA domain (25, 26, 27). While the coiled coil provides structure in PAN and Rpt1–6, in Mpa it has the additional function of recognizing Pup. There is only one OB domain in PAN and Rpt1–6, but there are two in Mpa, perhaps to expand the axial channel depth to match the N-terminal peptide of Pup that is first threaded into the Mpa channel to initiate peptide unfolding by the AAA domain (14).

**Figure 1:**
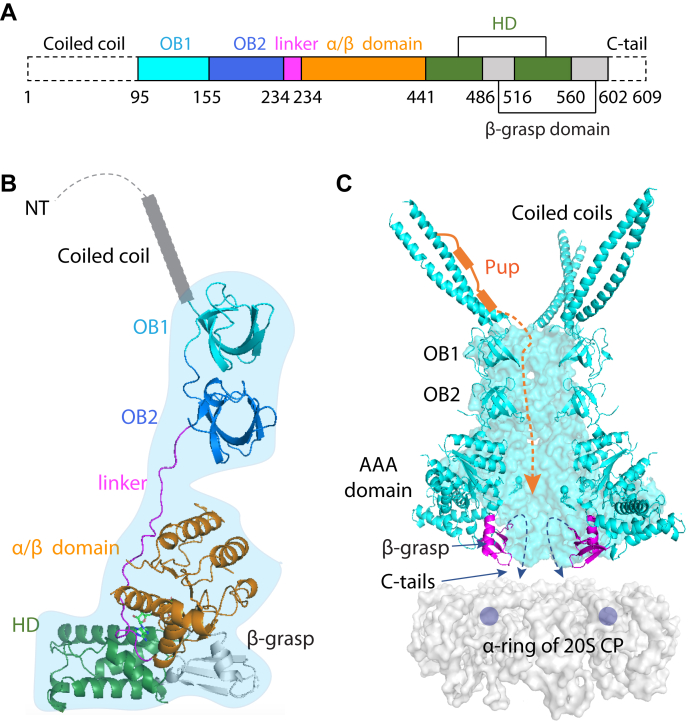
**The sixfold symmetric Mpa hexamer in the ADP-bound state is unable to bind to the 20S core particle (CP).***A*, domain organization of Mpa. *B*, the structure of an Mpa protomer determined in this study. *C*, Mpa hexamer in the ADP-bound symmetrical ring conformation (Protein Data Bank ID: 5KWA). The front two protomers are removed to show the vertical substrate path through the axial channel of the ATPase. Because the last resolved residue, Thr-601, is tugged inside the central chamber, the last eight residues (including the C-terminal GQYL motif) are partially hidden in the axial channel and cannot reach to the binding pocket on the top α-ring of the 20S CP, which is shown as a *gray* surface.

How an Mpa hexamer docks onto a 20S CP to open up the substrate entry gate is currently unknown, and no one has successfully reconstituted robust degradation of a pupylated protein by WT Mpa and 20S CPs. However, we and others have shown that another bacterial proteasome activator, PafE or bacterial proteasomae activator, can robustly stimulate the degradation of native substrates by the Mtb 20S CP, remarkably in the absence of ATP (28, 29). PafE forms 12-fold symmetric rings that also use GQYL motifs to activate degradation (30). Activation occurs when GQYL sequences insert in between α-subunits that gate Mtb 20S CPs (28, 30). Although Mpa also requires this GQYL motif, a previous crystal structure showed that docking is prohibited by the presence of a β-grasp domain unique to Mpa and its orthologs; in the apo form or in the ADP-bound form, Mpa forms a closed ring with the β-grasp domains forming a constriction around the central channel, preventing the C-terminal GQYL motifs from interacting with 20S CPs (20) (Fig. 1*C*). Therefore, a major challenge to the field is understanding how Mpa interacts with 20S CPs to activate protein degradation.

To gain a better understanding of Mpa dynamics, we used cryo-EM to study this protein in complex with a pupylated substrate. We demonstrate that Pup and ATP stimulate Mpa ATPase activity and binding to “open-gate” 20S (20S_OG_) CPs. From a 4-Å resolution cryo-EM structure in the presence of a pupylated substrate, we show that an Mpa hexamer binds one ATP and two ADP molecules to form a gapped-ring structure that can better interact with a 20S CP. We also show that the linker between the OB domain and AAA domain is essential for normal Mpa function *in vivo*.

## Results

### Pupylated substrate binding to Mpa stimulates ATPase activity *in vitro*

**Figure 2:**
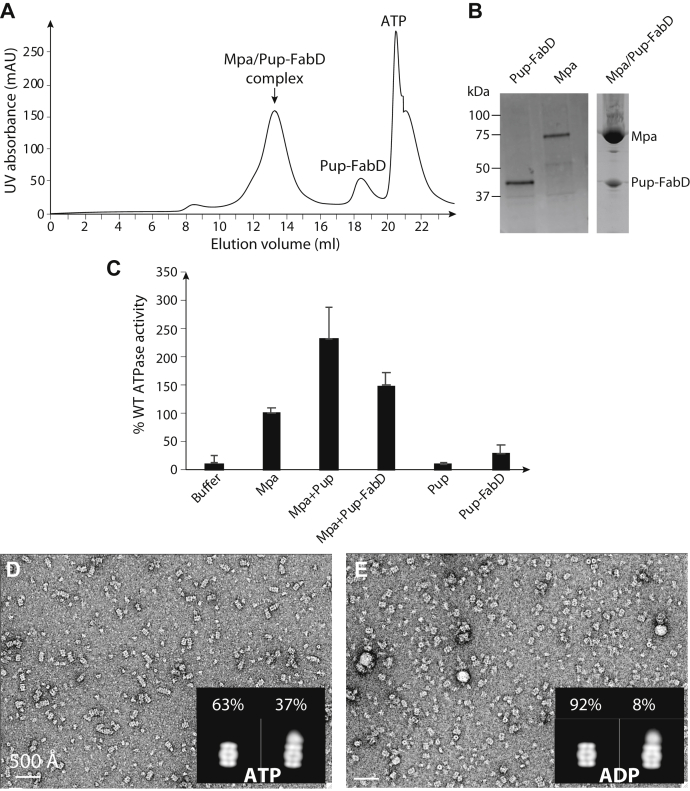
**Mpa interacts with *Mycobacterium tuberculosis* (Mtb) 20S proteasome core particle in the presence of ATP and Pup–FabD.***A*, gel filtration profile of the Pup–FabD–Mpa complex. *B*, SDS-PAGE gel of purified Mpa, Pup–FabD fusion, and the complex eluted gel filtration peak volume (13.2 ml). *C*, ATP hydrolysis activity of Mpa alone or in the presence of Pup or Pup–FabD. *D* and *E*, negative-stain electron micrographs of the mixtures of purified Mpa, Pup, and the Mtb 20S_OG_ CP in the presence of ATP (*D*) or ADP (*E*). Inserted in each panel are 2D class averages of 20S_OG_ CPs alone (*left*) and with Mpa cap (*right*) in the presence. Percentages of the capped *versus* uncapped 20S_OG_ CP are shown above the averages. 20S_OG_, open-gate 20S; FabD, malonyl CoA-acyl carrier protein transacylase; Pup, prokaryotic ubiquitin-like protein.

In *Escherichia coli*, we separately produced and purified the full-length Mtb Mpa and Pup–malonyl CoA-acyl carrier protein transacylase (FabD), in which Pup was translationally fused to the N terminus of FabD. We combined Pup–FabD and Mpa in the presence of 3 mM ATP and isolated a complex by gel filtration (Fig. 2*A*; see Experimental procedures section). An SDS-PAGE gel analysis of the peak fraction demonstrated the formation of the intended complex (Fig. 2*B*). We next examined the substrate-stimulated ATPase activity of Mpa *in vitro*. We added either purified Pup or Pup–FabD to purified Mpa, and by the malachite green phosphate assay, we found that both Pup and Pup–FabD stimulated the ATP hydrolysis activity of Mpa by a factor of 1.5 to 2 (Fig. 2*C*).

### ATP stimulates Mpa binding to Mtb 20S_OG_ CPs

We hypothesized that ATP binding may position Mpa hexamers into a conformation more suitable for interfacing with 20S CPs. To test this hypothesis, we used negative-staining EM of 20S_OG_ CPs (7), as previous work showed that deletion of the N-terminal octapeptides of the proteasome core α-subunits opens the substrate entry gate of the α-ring of the 20S CPs and facilitates or strengthens interactions with known proteasome activators (20, 31). 20S_OG_ CPs have increased proteolytic activity toward small peptides (32) and facilitates binding with Mpa proteins with artificially extended C termini (20). We mixed purified Pup, Mpa, and the 20S_OG_ CPs in the presence of either 3 mM ATP or 3 mM ADP and performed negative-stain EM of the reaction mixtures. We focused our analysis on the side views of the 20S_OG_ CPs because it was easier to detect the presence of Mpa in these views. We found that only 8% of 20S_OG_ CPs bound to Mpa hexamer in the presence of ADP, as compared with 37% of the 20S_OG_ CPs bound in the presence of ATP (Fig. 2, *D* and *E*). This result suggested that ATP binding is required for Mpa to interface with 20S CPs. The requirement of ATP for 20S CP association has also been observed for the archaeal ATPase PAN and the eukaryotic Rpt1–6 (33, 34). However, under the conditions we used, Mpa associated with 20S_OG_ CPs was not well ordered, as indicated by the blurred density at one or both ends of the 20S CPs. This unstable binding between Mpa and 20S_OG_ CPs prevented a high-resolution cryo-EM structural analysis of the Mpa–20S_OG_ CP complex.

### Mpa structure in the presence of ATP is drastically different from ADP-bound Mpa

**Figure 3:**
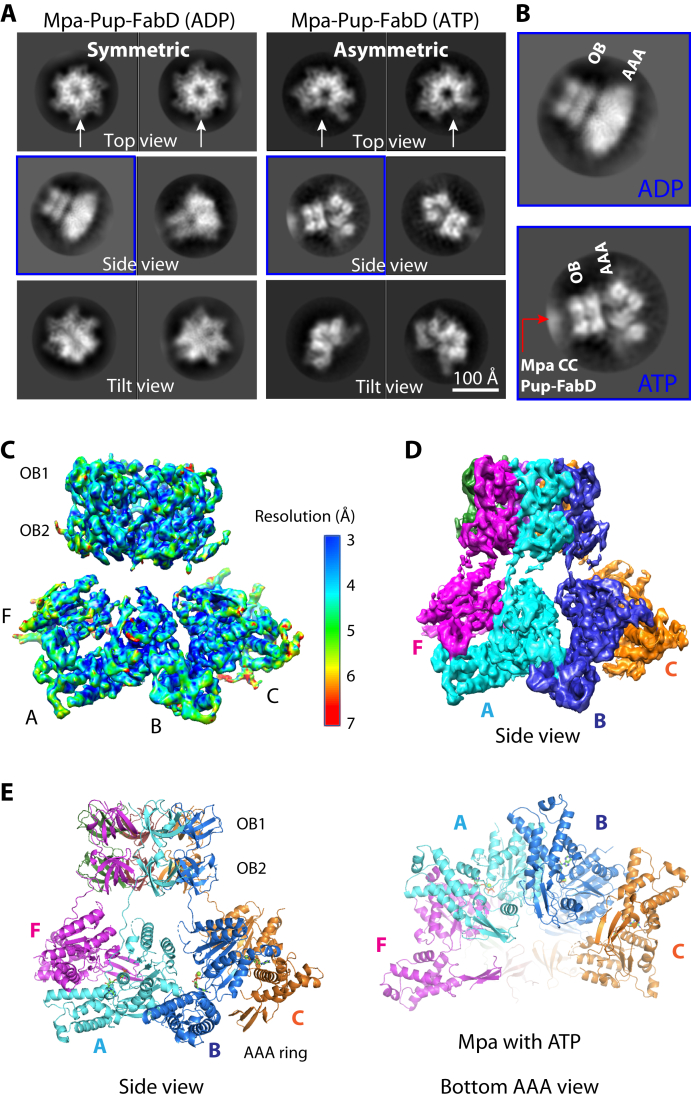
**Cryo-EM structure of Pup–FabD–bound Mpa hexamer in the ATP state.***A*, representative 2D class averages of Pup–FabD–Mpa complex in the presence of ADP (*left*) or ATP (*right*). The *white arrows* point to the complete rings in the ADP form and the gapped rings in the ATP form. *B*, enlargement of the average side views of Mpa in the ADP and ATP states. The *red arrow* points to a fuzzy density from Pup–FabD bound to the Mpa coiled coils. *C*, local resolution map of cryo-EM 3D map of Mpa hexamer in the ATP state. *D*, side view of the surface-rendered 3D map. *E*, atomic model of Mpa hexamer in the ATP state shown in a side and bottom view. Subunits are colored individually. 20S_OG_, open-gate 20S; FabD, malonyl CoA-acyl carrier protein transacylase; Pup, prokaryotic ubiquitin-like protein.

The increased binding between Mpa and 20S_OG_ CPs in the presence of ATP suggested that Mpa may undergo a conformational change that enables a stronger interaction with 20S_OG_ CPs. We therefore carried out a cryo-EM analysis on the Pup–FabD:Mpa complex assembled in the presence of 3 mM ATP and isolated by size-exclusion chromatography (Fig. 2, *A* and *B*). We added either 5 mM ATP or 5 mM ADP to the purified complex before preparing the cryo-EM grids. Imaging of a frozen-hydrated specimen showed monodispersed particles (Fig. S1). Although Pup–FabD was present in the *in vitro* assembled complex, we did not observe a well-defined density except for a cloudy patch at its expected location, which is above the OB rings in the averaged side views of the complex particles (Fig. 3, *A* and *B*). FabD is covalently but flexibly linked to Pup, and Pup binds to one of the three pairs of the N-terminal coiled-coil domains of the Mpa hexamer (14). The Mpa N-terminal coiled coil is linked to the stable OB ring *via* a highly flexible hinge (Pro-97−Pro-98). The presence of these two flexible hinges between FabD and the main body of an Mpa hexamer explains the absence of a clear Pup–FabD density, despite the approximately one Pup–FabD per one Mpa hexamer-binding stoichiometry. In a previous crystal structure of the Mpa hexamer with the AAA domain truncated, the coiled coils are visible because of two Mpa hexamers interacting with each other using their respective coiled-coil domains (14). Because Pup–FabD was not observed in the averaged EM images, we will refer to this complex simply as “Mpa” from here on.

Several side views of the Mpa hexamer in the presence of ADP compared with those in the presence of ATP showed that the OB rings are identical, but the lower AAA tiers are very different in the two nucleotide states (Fig. 3*A*). In averaged top view images, Mpa in the presence of ADP formed symmetrical and closed-ring structures, consistent with our previous crystal structure (20). However, with ATP, the Mpa ring was missing a substantial amount of hexamer density, suggesting that a portion of an Mpa hexamer becomes disordered (Fig. 3*A*). A closer look at the side views of Mpa in two different nucleotide states revealed that major changes occur in the AAA tier (Fig. 3*B*). Because the structural features of class averages of Mpa in the ADP state in solution agree with the 3.0-Å crystal structure of the symmetrical Mpa hexamer in the same state (20) (Figs. 1*D* and 3*A*), we did not pursue a cryo-EM determination of the ADP-bound Mpa structure in solution.

### The AAA domain of an Mpa hexamer forms a gapped ring in the presence of ATP

After extensive 2D and 3D classifications, we selected some 350,000 particle images of Mpa in ATP for final 3D reconstruction, leading to a 4.0-Å resolution 3D map (Fig. S1; Table S1). The resolution could not be improved further because a portion of the structure at the AAA tier is somewhat flexible. In the new Mpa structure, as we expected from the 2D averages, the coiled-coil domains are entirely missing and the two OB domains of each protomer form the rigid OB double ring, but only four of the six AAA domains are resolved in the hexamer (Fig. 3, *C* and *D*; Movie S1). We further examined Mpa architecture in the presence of the slowly hydrolyzable ATP analog ATPγS and the nonhydrolyzable analog adenylyl-imidodiphosphate (AMP-PNP). Based on 2D class averages and the low-to-medium resolution cryo-EM 3D maps, we found the overall architecture essentially the same as in the presence of ATP (Fig. S2, *A* and *B*). We expected that ATP hydrolysis of the system resulted in Mpa being in a mixed-nucleotide state. Therefore, Mpa hexamers in solution, whether in the presence of ATP, AMP-PNP, or ATPγS, seem to have all six coiled-coil domains largely flexible, with all six double OB domains forming the stable double OB ring, but with the AAA domains of two Mpa protomers being highly flexible and invisible in the 3D reconstructions.

**Figure 4:**
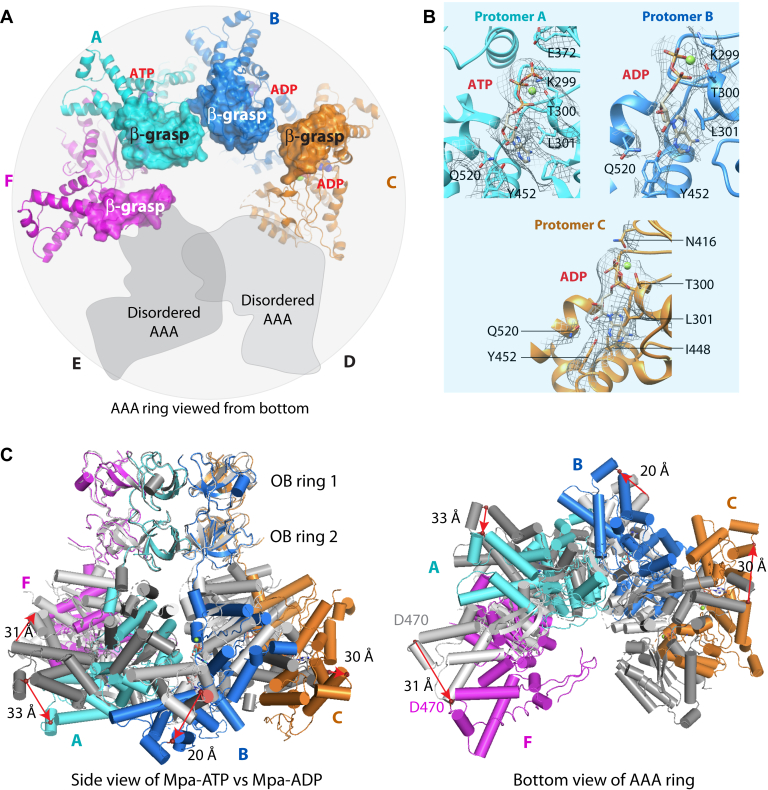
**Comparison of ATP- and ADP-bound Mpa structures.***A*, the β-grasp domains do not form a constriction in Mpa structure in ATP (*bottom view*). The β-grasp domains are in surface view, and two disordered AAA domains shown as *gray* shapes. Nucleotides are in *spheres*. *B*, enlarged views of the nucleotide-binding pockets in protomers A, B, and C, superimposed with the bound nucleotide electron densities in *gray surface meshes*. Key residues are shown in *sticks* and with their densities in *meshes*. *C*, comparison of Mpa hexamer in the presence of ADP (*gray*) or ATP (*color*). Structures are aligned by OB domain and depicted as *cylinders*. Residue D470 in each subunit is shown as *red sphere* to illustrate domain movement. AAA, *A*TPase *A*ssociated with various *A*ctivities; OB, oligonucleotide-/oligosaccharide-binding.

The flexibility of the two AAA domains appears to create a large gap in the AAA ring of the Mpa hexamer (Fig. 4*A*). The densities for the β-grasp domains of protomers A and B were fully visible, but the corresponding densities in protomers C and F were only partially present. Therefore, we could only include partial β-grasp domains in protomers C and F. Interestingly, we found that only the nucleotide-binding site in protomer A was occupied by ATP; the protomers B and C sites were occupied by ADP; and the nucleotide density in protomer F site was weaker and likely in the apo state. Therefore, an Mpa hexamer in the presence of 5 mM ATP is in a mixed nucleotide state, bound to both ATP and ADP. This gapped flexible ring architecture contrasts with our previous crystal structures that show six AAA domains forming a stable and closed flat ring in the fully ADP-bound state.

Superimposing the shared double OB ring of the cryo-EM structure and the ADP-bound crystal structure revealed detailed changes of individual AAA domains in these two states (Fig. 4*B*). Overall, the four AAA domains move as individual rigid bodies; they rotate and expand counterclockwise when viewed from bottom C-terminal face. However, each AAA has distinct repositions. Specifically, if we use Asp-470 in the small helical subdomain of the AAA domain as a landmark, the AAA domains of protomers A, B, C, and F in the cryo-EM structure shift 33, 20, 30, and 31 Å, respectively, from their positions in the ADP-bound state. Furthermore, only protomer C moves inplane and outward, whereas protomers A and B move downward, and protomer F moves slightly upward. Finally, the movement includes both shift and rotation (Fig. 4*B*). It is these compound movements that transform the AAA ring from a closed and six-fold symmetric ring in the ADP-bound state into a gapped ring in the presence of ATP.

### The short α-helix in the OB−AAA linker uncoils to allow AAA domain movement

**Figure 5:**
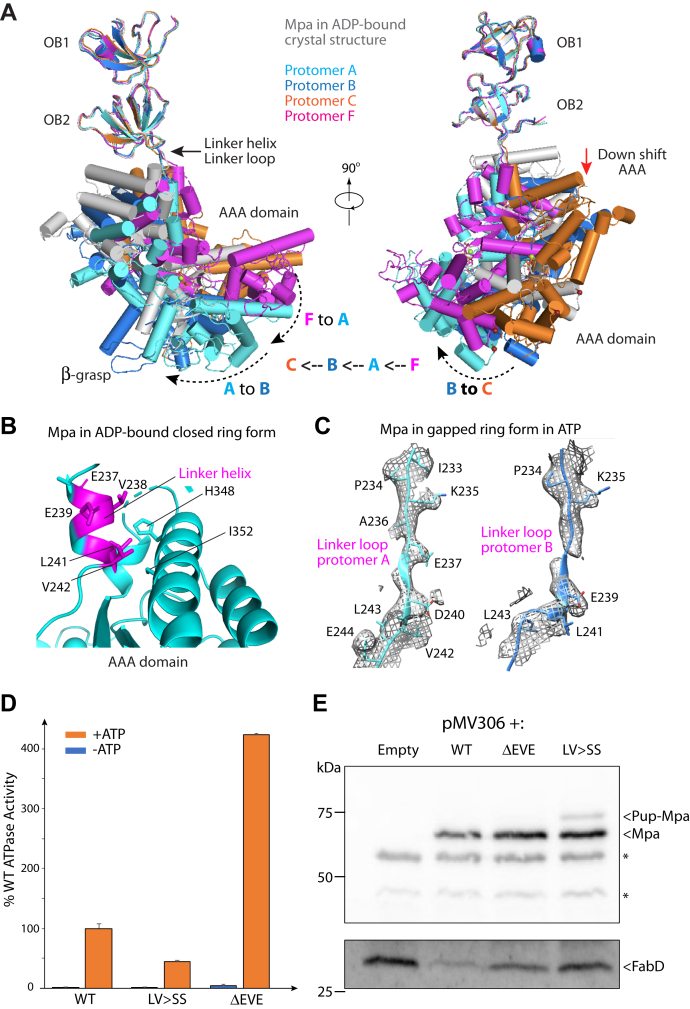
**Linker loop between OB and AAA domain is essential for function.***A*, alignment of Mpa protomers A, B, C, and F individually with an Mpa protomer in the ADP-bound crystal structure (Protein Data Bank ID: 5KWA). *B*, the linker forms a short helical coil in the ADP-bound ring form of Mpa. *C*, the linkers are in extended loop configurations in the ATP form. *D*, ATP hydrolysis activity of purified WT and two mutant Mpa proteins *in vitro*. *E*, substrate degradation as mediated by the WT or mutant Mpa proteins in Mtb. *Top*, antibodies to Mtb Mpa detect both WT and mutant Mpa. In some experiments, pupylated Mpa can also be observed, as indicated. *Bottom*, antibodies to Mtb FabD show that both mutant Mpa have more accumulated FabD than in the *mpa* mutant complemented with WT *mpa*. ∗ indicates crossreactive species that act as a loading control. AAA, *A*TPase *A*ssociated with various *A*ctivities; FabD, malonyl CoA-acyl carrier protein transacylase; Mtb, *Mycobacterium tuberculosis*; OB, oligonucleotide-/oligosaccharide-binding.

We next individually superimposed the four complete Mpa protomers in ATP with an Mpa protomer of the ADP-bound crystal structure. We found that the large AAA domain movement is coupled with conformational changes of their respective linker regions between the double OB domains and the AAA domains (Movie S2). During the ADP state to ATP state transition, all four Mpa AAA domains moved downward by a few angstroms (Figs. 4*C* and 5*A*). This movement was made possible by changes in the 26-residue long linker loop, from Arg-232 to Ser-258. The ADP-bound crystal structure showed this linker has a one-turn α-helix, stabilized by the α/β subdomain of the AAA domain (Fig. 5*B*). The short α-helix detaches from the α/β subdomain and uncoils to extend the linker, thereby enabling the downward movement of the AAA domain in the presence of ATP (Fig. 5*C*). Concomitant to the downward shifts, the four AAA domains also rotate about the long axis of the hexamer, transforming from the sixfold symmetric structure in the ADP state to the spiral structure. The structural transitions suggest a functional role of the linker loop.

Stabilization of the short coil of the linker in the ADP state involves multiple interactions of 237-Glu–Aal–Glu-239 and 241-Leu–Val-242 in the linker with the α/β subdomain of the AAA domains. This linker region is highly conserved among bacterial proteasome ATPases (Mpa/ATPase forming ring-shaped complex) but less so in PAN and the eukaryotic proteasomal ATPases. We hypothesized that destabilizing the helical coil would interfere with the ability of Mpa to undergo the nucleotide binding and hydrolysis cycle, potentially compromising its function. Thus, we made two mutants: one with a deletion of amino acids 237 to 239 (ΔEVE) and the other replacing residues 241 to 242 with two serines (LV > SS). We found that purified mutant Mpa LV > SS had about half of the WT ATPase activity, but unexpectedly, the ATPase activity of ΔEVE mutant protein was four times higher than that of WT Mpa (Fig. 5*D*). However, both mutations reduced the degradation of a model proteasome substrate, FabD (Fig. 5*E*). Mpa is also an established Pup–proteasome substrate and occasionally appeared accumulated in the loop mutant strains. Therefore, our analysis has revealed that this linker region in Mpa is essential for robust protein degradation.

### The Mpa pore loops form a left-handed staircase in the presence of ATP

**Figure 6:**
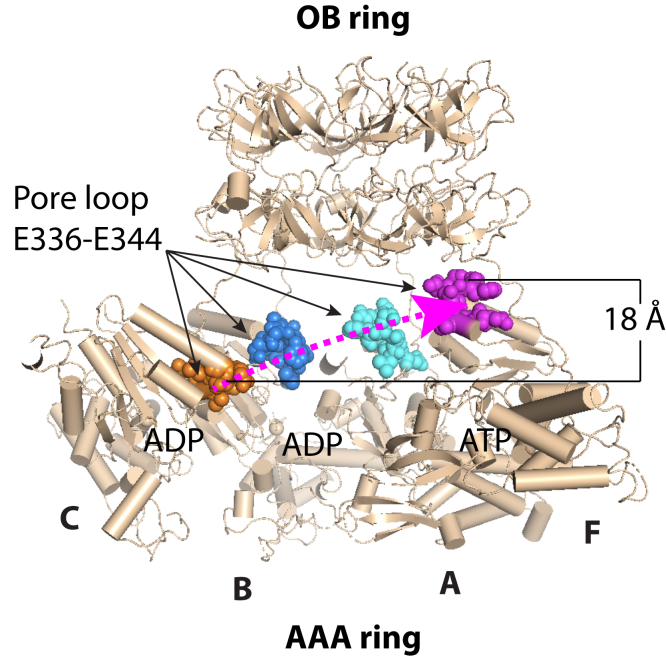
**Substrate pulling pore loops form a left-handed spiral.** Pore loops of the upper chambers adopt a staircase arrangement in the ATP-bound Mpa open conformation. Pore loops of chains F, A, B, and C in the structure labeled as *magenta*, *cyan*, *blue*, and *orange spheres*. The *dashed magenta curve* shows the four loops spiral upward by about 18 Å, with each loop stepping up by about 6 Å.

In the ADP-bound Mpa hexamer crystal structure, the six pore loops (E336 to E344) form a planar ring around a 26-Å-wide opening in the central chamber (20). Phe-341 in the middle of pore loop is the predicted primary site where the pore loops contact and pull on the substrate. The importance of this pore loop was demonstrated previously by an F341A mutation that abolished Pup- and Mpa-mediated protein unfolding (15). In the presence of ATP, the pore loops of the four observed AAA domains of Mpa arranged into a left-handed staircase, rising about 6 Å per protomer (Fig. 6 and Fig. S3). Given that the pore loops of the Rpt1–6 hexamer in the human 26S proteasome are arranged into a right-handed staircase (26) and many other AAA protein unfoldases require right-handed configurations for function (35), we propose that the Mpa structure represents an intermediate state, and that its pore loops switch to a right-handed staircase upon stable interaction with 20S CPs.

## Discussion

**Figure 7:**
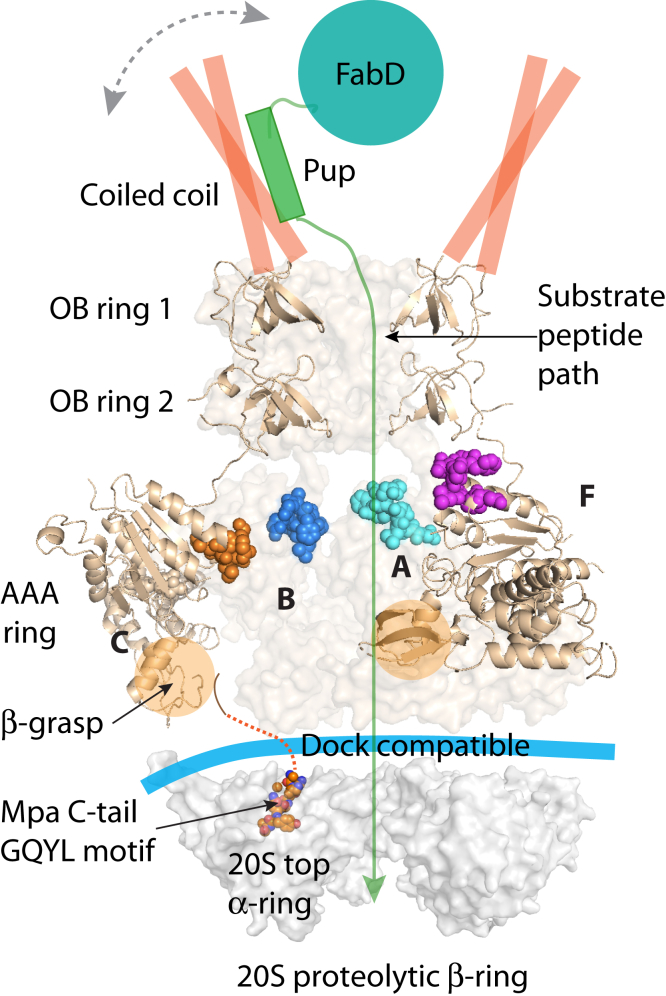
**A hypothetic model for Mpa docking on the 20S CP.** We propose that the C termini of Mpa hexamer in ATP are fully exposed, in a configuration compatible with docking with 20S proteasome (Protein Data Bank ID: 5THO; only the top α-ring is shown). The *dashed red line* indicates the path toward the activating sites in a PrcA ring. The *horizontal thick cyan curve* indicates the compatible docking interface between an Mpa hexamer and 20S CP. The C-tail GQYL of one Mpa is shown in *spheres*; its insertion in the 20S CP is based on the PafE-20S CP structure and other biochemical evidence. Pup–FabD is shown as sketch as it is not visible in the cryo-EM structure. The *gray curve with arrowheads* at both ends indicates high flexibility of the Mpa coiled coil and its associated Pup–FabD. CP, core particle; GQYL, Gly–Gln–Tyr–Leu; PrcA, proteasome core α-subunits; Pup, prokaryotic ubiquitin-like protein.

In this study, we show that in solution, Mpa in the presence of ATP forms a flexible and gapped ring, a configuration that is more compatible with binding to Mtb 20S_OG_ CPs than structures we previously determined by X-ray crystallography. This observation may partly resolve the puzzle raised by the reported Mpa crystal structures in which the C-terminal–activating GQYL motifs are largely hidden inside the hexameric chamber, preventing interactions with the α-ring of Mtb 20S CPs. Although they are not resolved in our structure, we predict that the C-terminal GQYL motifs are more exposed in this gapped ring conformation, as the β-grasp domains do not form a constriction (Fig. 4*A*). The presence of two flexible AAA domains along with other movements could potentially allow for the repositioning of the β-grasp domains. This movement would then position the C-terminal GQYL sequences closer to the 20S CP-activating pockets to stimulate degradation (Fig. 7).

We observed three nucleotide states in one Mpa hexamer: protomer A bound to ATP, both protomers B and C bound to ADP, and protomer F in apo form, largely absent of a nucleotide. The mixed nucleotide-binding pattern agrees with many recently determined structures of AAA protein unfoldases (36). For example, in the ATPγS-bound human 26S proteasome structure, four Rpt subunits bind to ATPγS and two Rpt subunits are in apo form (37). In another human 26S structure determined in the presence of 5 mM ATP, five Rpt subunits have ATP bound and one has ADP bound. A cryo-EM study of PAN revealed the binding of four protomers to ATP, one protomer to ADP, and one protomer in apo form (25). Based on these considerations, we suggest that the previously observed all-apo and all-ADP-bound states of Mpa are not functional (20). The crystallization-induced single-nucleotide states (all-apo, all-ADP, or all-ATP) have also been observed in structures of the HslV protease–associated ATPase HslU (38, 39).

Mpa in the presence of substrate and ATP adopted an open ring conformation in which the peptide translocation loops were arranged in a left-handed manner and the C-terminal β-grasp domains were shifted outward. A similarly gapped ring, in fact a left-handed spiral, has been reported for several hexameric AAA+ ATPases. For example, Mtb ClpB disaggregase is an open and left-handed spiral in the presence of ADP or AMP-PNP before engaging peptide substrate. But, ClpB switches to a largely closed ring with the pore loops arranged in a right-handed spiral upon engaging a substrate peptide in the presence of ATP (40). The *Schizosaccharomyces pombe* Abo1 hexamer, a histone chaperone ATPase, is a flat ring in the ADP-bound state, but it switches to a right-handed spiral in the presence of ATP and histone 3 tail (41). The yeast Mcm2–7 hexamer, a replicative double-stranded DNA helicase core, is an open and left-handed spiral in the presence of saturating AMP-PNP (42). However, in the functional Cdc45−Mcm2–7−GINS helicase engaged with a forked DNA substrate in ATPγS, the Mcm2–7 hexamer switches to a largely closed ring with the DNA translocation loops in a right-handed staircase (43).

We therefore suggest that the cryo-EM structure of Mpa represents a functional intermediate state that is more compatible with substrate and 20S CP binding but is not yet in a substrate unfolding–competent conformation. This idea is supported by the fact that even in the presence of ATP, intact Mpa and 20S CPs fail to robustly degrade pupylated proteins; even with an artificial extension of the GQYL motifs of Mpa and ATP, we (and others) observe degradation only using 20S_OG_ CPs (20, 31). We expect that additional conformational changes are required when Mpa tightly and stably associates with the 20S CPs to form functional complexes. Thus, we propose that additional factors are needed for the robust interaction of Mpa with WT 20S CPs, a hypothesis we are actively testing in our laboratories.

Given that the Pup−Mpa−proteasome system is essential for Mtb to survive in a host, several inhibitors targeting Mtb proteasomes have been developed (44, 45, 46, 47). Therefore, a detailed mechanistic understanding of the bacterial Pafs is not only important for proteasomal biology but also may be important for antituberculosis drug development.

## Experimental procedures

### Molecular cloning, protein expression, and purification

In our study, the C-terminal His-tagged Mtb full-length Mpa (1–609), Pup (1–64), and Pup–FabD were used to transform competent BL21(DE3) *E. coli* for protein expression. Transformed BL21(DE3) strains were cultured in LB medium with 100 μg/ml kanamycin at 37 °C for 4 h. When an absorbance at 600 nm reached 0.8 to 1.0, cells were induced by adding IPTG at a final concentration of 0.2 mM and then cultured at 16 °C overnight to express proteins. Bacterial cells were collected by centrifugation at 4000*g* for 20 min at 4 °C, and fresh cells were immediately resuspended in buffer A (25 mM Tris–HCl, pH 8, 200 mM NaCl, and 10 mM MgCl_2_) supplemented with an EDTA-free protease inhibitor cocktail (Roche). Cells were lysed by passing through a French press once at 1800 bars, followed by centrifugation at 45,000*g* for 45 min at 4 °C. Supernatant was collected and loaded onto a 5-ml Ni-nitrilotriacetic acid (Qiagen) pre-equilibrated with buffer A and eluted with an imidazole step gradient. Elution fractions containing Mpa were immediately concentrated and subjected to a gel-filtration purification step (Superose increase 6; GE Healthcare) in buffer A. Fractions corresponding to Mpa peak were pooled for further use. Protein concentration of Mpa at this stage was about 12 mg/ml. The same purification strategy was applied to purify Pup and Pup–FabD. The only difference is that Pup was run through Superdex 75 10/300 GL column, and Pup–FabD was run through the Superdex 200 Increase column, because of their different molecular weights. The protein concentration of Pup and Pup–FabD after gel filtration was about 4.0 and 2.0 mg/ml, respectively.

We next incubated 1 M equivalent of the Mpa hexamer with 2 M equivalents of Pup–FabD in 3 mM ATP on ice for 10 min, or we incubated 1 M equivalent of the Mpa hexamer with 3 M equivalents of Pup in 3 mM ATPγS or AMP-PNP on ice for 1 h. The sample was gel filtered through Superose 6 Increase (GE Healthcare), which was pre-equilibrated with buffer C (20 mM Tris–HCl, pH 8.0, 200 mM NaCl, 5 mM MgCl_2_, and 10 μM ATP). The final target peak was assessed by SDS-PAGE gel to validate complex assembly.

### Purification of 20S_OG_ CPs

The Mtb 20S_OG_ was purified as previously described (7). Briefly, competent BL21(DE3) transformed with the His-tagged Mtb 20S CPs were cultured in LB medium supplemented with 34 μg/ml chloramphenicol at 37 °C and grown to an absorbance of 0.5 to 0.7, and then protein expression was induced by adding 0.3 mM IPTG at 37 °C and cells were grown for 16 h. Cells were harvested by centrifugation at 4000*g* for 20 min. Collected frozen cells were lysed in buffer F containing 20 mM Tris–HCl, pH 8, 200 mM NaCl, and 5 mM MgCl_2_. After centrifugation, protein was purified from the supernatant using Ni-nitrilotriacetic acid affinity chromatography. Elute was concentrated and subjected to a final gel filtration purification (Superose 6 Increase) in buffer F. Target fractions were pooled for further use. The protein concentration at this stage was about 2.4 mg/ml.

### Negative-staining EM of the Pup–Mpa–20S_OG_ complex

We incubated 2 M equivalents of Mpa hexamer with 3 M equivalents of Pup and 1 M equivalent of Mtb 20S_OG_ CPs with either 3 mM ATP or 3 mM ADP on ice for 40 min. For negative-staining EM, a 5-μl drop of diluted mixture was applied to a glow-discharged 300-mesh copper grid covered with a thin layer of carbon film. After removing excess solution by blotting with a piece of filter paper, sample grid was stained with two 5-μl drops of 2% (w/v) uranyl formate aqueous solution. Excess stain solution was blotted, and grids were left to air dry. Negative-stained EM grids were imaged at room temperature in a Tecnai G2 Spirit electron microscope (FEI) operated at 120 kV using low-dose procedure. Images were recorded at a magnification of 49,000× and a defocus value of 1.2 μm on an Eagle charge-coupled device camera. All images were binned (4096 pixels) to obtain a final pixel size of 2.14 Å on the specimen level. We manually picked 1000 particles to generate 2D averages as template for subsequent automatic particle picking. A total of 232,271 particle images in the ATP form and 27,680 particle images in the ADP form were automatically picked. 2D classification was performed in RELION-3.1 from the Sjors Scheres lab (48). We selected only side views of proteasome particles with and without bound Mpa to calculate the occupancy.

### Cryo-EM

We used the gel filtration–purified Pup–FabD–Mpa complex for structural analyses. Before preparing cryo-EM grids, we preincubated the sample with either 5 mM ATP for 5 min, or 3 mM ATPγS for 30 min, or 3 mM AMP-PNP for 30 min, on ice to form the intended complex structure. The samples were diluted to 10 μM for cryo-EM grid preparation. Samples (3 μl) were deposited onto freshly glow-discharged holey carbon grids (Quantifoil Cu R2/1; 300 mesh) and blotted using a Thermo Fisher Scientific Vitrobot Mark IV with standard Vitrobot filter paper. Blotting time was set to 3 s, blotting force was set to 4, and blotting was done under 100% humidity at 9 °C. The blotted grids were flash frozen in liquid ethane and stored in liquid nitrogen.

Cryo-EM dataset of Mpa incubated in ATP was collected in a 300-kV Thermo Fisher Scientific Titan Krios electron microscope operated at a nominal magnification of 130,000× with defocus values from −1.0 to −1.8 μm on a Gatan K3 detector with a pixel size of 0.826 Å per pixel at the sample level. Dose rate was 1.5 electrons per Å^2^ per second per frame, and 40 frames were recorded in a movie. We recorded 8152 raw movie micrographs. Cryo-EM datasets of Mpa incubated in ATPγS and Mpa incubated in AMP-PNP were collected in the Titan Krios on a Gatan K2 camera. We recorded 3250 raw movie micrographs for Mpa–ATPγS and 4250 raw movie micrographs for Mpa–AMP-PNP. Micrographs were recorded in super-resolution counting mode at a nominal magnification of 130,000, resulting in a physical pixel size of 1.09 Å per pixel. Defocus values varied from −1.2 to −2.5 μm. Dose rate was 10.2 electrons per pixel per second. Exposures of 6 s were dose fractionated into 30 subframes, leading to 0.2 s exposure time for each frame.

### Image processing and 3D reconstruction

All movie micrographs were motion corrected using the UCSF MotionCorr-2 program (49). Contrast transfer function parameters of each aligned micrograph were calculated using CTFFIND-4.18 from the Nikolaus Grigorieff lab (50). All remaining steps were performed using RELION-3 (48). We divided the Mpa-ATP dataset into two subsets of 2654 and 5498 micrographs. Templates for automatic picking were generated from a 2D average of about 1500 manually picked particles. The 2D classification and further 3D classification using subsets were performed to quickly remove the contamination and junk particles. For the Mpa–ATP complex, 345,023 particles belonging to the 3D class and having the best density features were selected for a second round of 3D classification. Refinement and postprocessing led to the final 3D reconstruction at an average resolution of 4.0 Å. For the Mpa–ATPγS and Mpa–AMP-PNP complexes, 451,000 and 699,051 particles, respectively, were selected for 3D classification, based on 2D class averages. A total of 68,202 and 108,491 particles, respectively, were selected for further 3D refinement, resulting in the 8.6-Å resolution 3D map for Mpa–ATPγS and 8.3-Å resolution 3D map for Mpa–AMP-PNP. The resolution of the 3D maps was estimated by gold-standard Fourier shell correlation at a correlation cutoff value of 0.143 (51). Local 3D resolution was calculated using the ResMap program from the Hemant Tagare lab (52).

### Atomic model building for the cryo-EM 3D map of Mpa

Atomic modeling was based on the published crystal structure of the ADP-bound Mpa (Protein Data Bank ID: 5KWA). We used the whole OB double ring and one AAA domain as two rigid bodies. The starting model was first rigid body docked into the cryo-EM 3D map of the 4-Å Mpa–ATP 3D map in UCSF Chimera (53). In the AAA tier, four copies of the AAA model were individually docked; then the derived model was manually adjusted and rebuilt in Coot (MRC Laboratory of Molecular Biology) (54). The resulting models were subjected to real-space refinement using the phenix.real_space_refine in the PHENIX program by the Paul Adams lab (55). The quality of the final model was assessed using Phenix comprehensive validation program (55). Because the resolutions of the 3D maps of Mpa in ATPγS and AMP-PNP were low, only rigid-body manual docking was performed. The resulting models fit well with the surface envelopes. No refinement was carried out. Structural figures were prepared using PyMOL (Schrödinger, LLC) and UCSF Chimera.

### ATPase assays

The ATPase activity of Mpa, with or without Pup or Pup–FabD, was determined by measuring the production of inorganic phosphate using the Malachite Green Phosphate Assay Kit (Sigma). Assays were started by adding 0.3 mM ATP to a solution (20 mM Tris–HCl, pH 8, 5 mM MgCl_2_, and 200 mM NaCl) containing 2 μg WT Mpa. Pup or Pup–FabD was added at 1.2 times the molar ratio of the Mpa hexamer in the reaction. The reactions were run for 30 min at 37 °C. Reaction products were collected every 10 min (80 μl) and added to 20 μl malachite green reagent in a 96-well plate. The amount of inorganic phosphate that was released was measured by absorbance change at 620 nm.

### Protein stability in *M. tuberculosis*

Sewing overlap extension PCR (56) was used to introduce mutations into Mtb *mpa*. We made a new *mpa* complementation plasmid with WT *mpa* (pHD300) to be isogenic to the constructs with introduced mutations. The primers used to amplify WT *mpa* (pHD300) were KpnImpa_pF: TAGGTACCCCACTACGCCTGTGGCTAGG and mpaH3R: TGAAGCTTCTACAGGTACTGGCCGAGGTTG (28). For “LV > SS” (pHD301) and “ΔEVE” (pHD302), we used MpaLV_SS_SOEF (GCCGAGGTAGAAGACTCGAGCCTGGAAGAGGTGCCG), MpaLV_SS_SOER (cGGCACCTCTTCCAGGCTCGAGTCTTCTACCTCGGC), MpaEVE_SOEF (CGCATCCCCAAAGCCGACCTGGTGCTGGAAG), and MpaEVE_SOER (CTTCCAGCACCAGGTCGGCTTTGGGGATGCG). All products were cloned into pMV306, which integrates at an *attB* site on the Mtb chromosome and used to transform MHD5, an *mpa*::MycoMarT7 null mutant (4).

Bacteria were grown in Middlebrook 7H9 medium to an absorbance at 580 nm of 1.7. Five absorbance-equivalent cell numbers were collected by centrifugation, washed in phosphate-buffered saline with Tween-80, and lysed in Tris–EDTA buffer using zirconia beads in a bead beater (Bio-Spec, Inc). Cell lysates were transferred to fresh tubes with SDS protein sample buffer and boiled for 10 min at 100 °C before removing them from the Biosafety Level 3 facility. Immunoblotting was done with antibodies to FabD–His_6_ and Mpa–His_6_, as previously described (57).

## Data availability

Cryo-EM 3D maps of the ATP-bound Mtb Mpa 4.0 Å have been deposited in the Electron Microscopy Data Bank under accession code EMD-23392, and the corresponding atomic model has been deposited in the Protein Data Bank under accession code 7LJF.

## Supporting information

This article contains supporting information.

## Conflict of interest

The authors declare that they have no conflicts of interest with the contents of this article.
