# Supplementary material for: The mycobacterial proteasomal ATPase Mpa forms a gapped ring to engage the 20S proteasome
Source: J Biol Chem. 2021 Apr 27;296:100713. doi: 10.1016/j.jbc.2021.100713 (PMC8142254; doi:10.1016/j.jbc.2021.100713)
Supplement: Supplemental Figures S1–S3 and Table S1 [file mmc1.pdf]

Supplemental materials for

The mycobacterial proteasomal ATPase Mpa forms a gapped ring to engage the 20S proteasome

Yanting Yin, Amanda Kovach, Hao-Chi Hsu, K. Heran Darwin, Huilin Li

This document contains

- 1 supplemental table and caption
- 3 supplemental figures and captions
- Legends for 2 supplemental movies

Supplemental Table 1. Cryo-EM data collection parameters and structure refinement statistics

	Mpa in ATP
**EMDB ID**	**EMD-23392**
**PDB ID**	**7LJF**
**Data collection**	
Microscope	FEI Titan Krios
Voltage (kV)	300
Detector	Gatan K3 Summit
Electron dose (e^-/A^2)	60
Pixel size (Å)	0.826
Defocus range (μm)	−1.0 to −1.8
**Reconstruction**	
software	RELION 3.1
Final particle #	345,023
Final Resolution (Å)	4.0
**Model composition**	
Protein chain	6
Protein residues	2007
Nucleotides	3
**R.m.s. deviations**	
Bond lengths (Å)	0.003
Bond angles (º)	0.688
**Ramachandran plot**	
Favored (%)	96.57
Outlier (%)	0.00
**Validation**	
Molprobity score	1.88
Rotamer outlier (%)	0.41
Clashscore	14.30

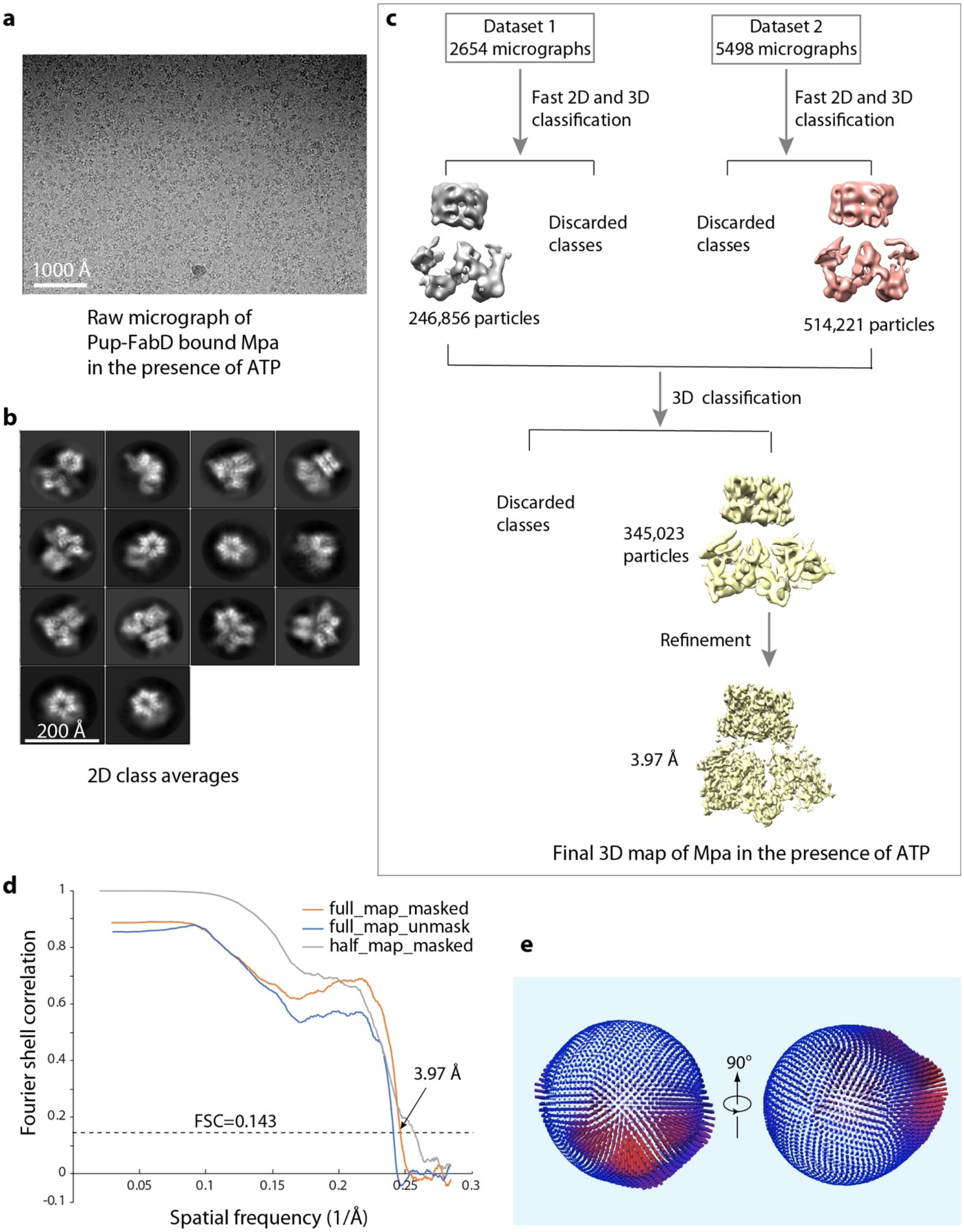

Supplemental Figure 1. Cryo-EM analysis of Pup-FabD bound Mpa in the presence of ATP. a) A representative electron micrograph. **b**) Selected 2D class averages. **c**) Workflow of cryo-EM data processing leading to the open conformation of Mpa in the presence of Pup-FabD and ATP. Pup-FabD is invisible in the map. **d**) Fourier shell correlations. **e**) Eulerian angle distribution of all particles used in the 3D reconstruction.

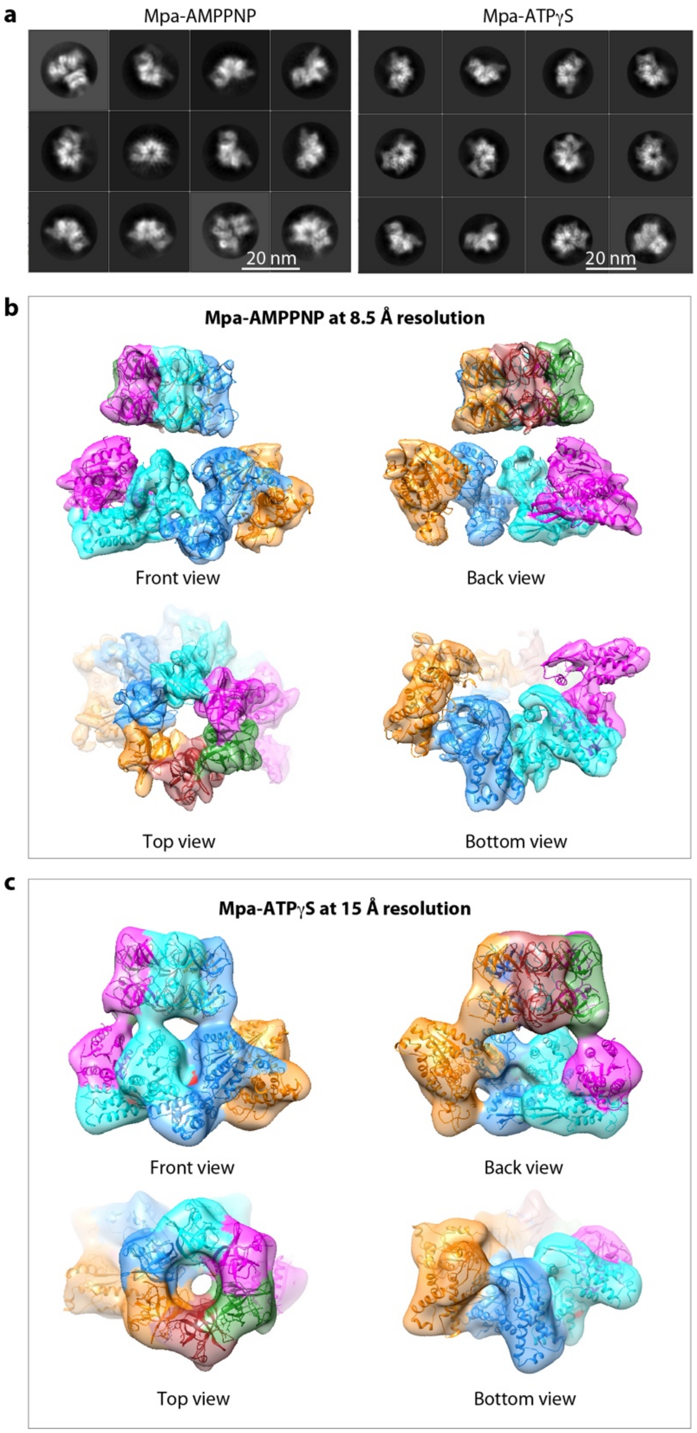

Supplemental Figure 2. Cryo-EM of Pup-FabD bound Mpa in the presence of AMPPNP or ATPγS. a) Selected 2D class averages of Pup-FabD bound Mpa in the presence of 5 mM AMPPMP (left panel) and 5 mM ATPγS (right panel). **b**) 3D map of Mpa in AMPPNP at 8.5-Å resolution superimposed with Mpa model in ATP. **c**) 3D map of the Pup-FabD bound Mpa in the presence of ATPγS at 15-Å resolution, superimposed with Mpa structure in ATP.

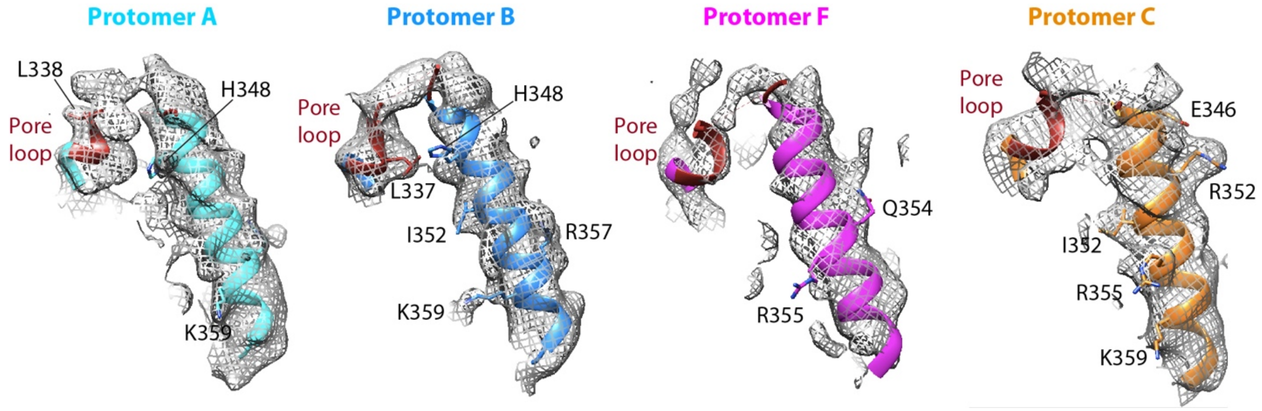

Supplemental Figure 3. Densities for the peptide translocation pore loops in Mpa in the presence of ATP. Each panel shows the pore loop density in mesh with atomic model of the pore loop and the associated α-helix superimposed.

LEGENDS FOR SUPPLEMENTAL MOVIES

Supplemental Movie 1. Cryo-EM structure of Mpa in the presence of ATP. The cryo-EM map is shown as transparent surface. The atomic model is show in cartoon with bound nucleotides shown in sticks.

Supplemental Movie 2. Atomic trajectory of Mpa structure morphing from the ADP-bound crystal conformation to the conformation observed by cryo-EM in the presence of ATP.
